# High‐precision iRT prediction in the targeted analysis of data‐independent acquisition and its impact on identification and quantitation

**DOI:** 10.1002/pmic.201500488

**Published:** 2016-06-28

**Authors:** Roland Bruderer, Oliver M. Bernhardt, Tejas Gandhi, Lukas Reiter

**Affiliations:** ^1^ BiognosysWagistrasse 25CH‐8952 SchlierenSwitzerland

**Keywords:** Bioinformatics, Chromatography, Data‐independent acquisition, iRT, Retention time alignment, Retention time normalization, Retention time prediction

## Abstract

Targeted analysis of data‐independent acquisition (DIA) data is a powerful mass spectrometric approach for comprehensive, reproducible and precise proteome quantitation. It requires a spectral library, which contains for all considered peptide precursor ions empirically determined fragment ion intensities and their predicted retention time (RT). RTs, however, are not comparable on an absolute scale, especially if heterogeneous measurements are combined. Here, we present a method for high‐precision prediction of RT, which significantly improves the quality of targeted DIA analysis compared to in silico RT prediction and the state of the art indexed retention time (iRT) normalization approach. We describe a high‐precision normalized RT algorithm, which is implemented in the Spectronaut software. We, furthermore, investigate the influence of nine different experimental factors, such as chromatographic mobile and stationary phase, on iRT precision. In summary, we show that using targeted analysis of DIA data with high‐precision iRT significantly increases sensitivity and data quality. The iRT values are generally transferable across a wide range of experimental conditions. Best results, however, are achieved if library generation and analytical measurements are performed on the same system.

**Abbreviations**
CV: coefficient of variation
DDA: data‐dependent acquisition
DIA: data‐independent acquisition
iRT: indexed retention time
RT: retention time
XIC: extracted ion chromatogram

## 1 Introduction

Liquid chromatography mass spectrometry (LC‐MS) is a powerful and widely used approach to identify and quantify proteins 1. Its unbiased and comprehensive nature makes it ideal to characterize proteins, detect differentially abundant proteins, to profile proteomes or to discover biomarkers 2, 3.

**Significance of the study**

Spectral libraries are crucial for the targeted analysis of DIA data. Accurate normalized RTs significantly contribute to the quality of a spectral library. Here, we investigate the influence of high‐precision iRT prediction on identification and quantitation and its stability across different systems. For this purpose, we varied nine different experimental factors that influence transferability of iRT. Our results will help to understand and improve the quality and transferability of spectral libraries.

An LC‐MS acquisition method that is currently rapidly evolving is data‐independent acquisition (DIA). DIA has been introduced to increase the depth of analysis and to overcome the limitations of reproducibility of classical data‐dependent acquisition (DDA or shotgun proteomics) 4, 5, 6, 7, 8, 9, 10, 11. Classically, DIA data was analyzed with shotgun search engines. Prior to shotgun searching, DIA data can also be preprocessed to reduce the complexity of MS2 spectra 6, 12, 13. These approaches have the advantage that no spectral library is required, the indexed retention time (iRT) concept is not relevant to those approaches. However for reasons of performance, the currently most widely used DIA method for proteomic experiments uses targeted analysis of DIA data based on spectral libraries 14. This method can be performed on most high‐resolution MS platforms of the newest generation and has been shown to outperform shotgun proteomics in comprehensiveness, quantitative reproducibility and precision 14, 15, 16, 17, 18, 19. The approach is especially well suited for quantitation of dozens to hundreds of samples from different conditions and states, where high reproducibility and data completeness becomes important.

In the targeted analysis of DIA data, the complexity of chimeric spectra originating from the large DIA precursor windows is handled by making use of a spectral library. Typically, spectral libraries are generated by performing shotgun proteomics on the sample type of interest 20, 21, 22. Peptide information is condensed, consensus relative fragment ion patterns are generated and retention times (RTs) are homogenized. Then, the spectral library is used to query the DIA mass spectra and generate extracted ion current chromatograms for precursor and fragment ions which build the basis for data analysis 14, 23. Because the expected elution time is typically available in the spectral library, the extracted ion current chromatograms can be limited to a specific window in the chromatogram where the peptide is expected to elute. The more accurate the elution time can be predicted the smaller the resulting window. A small window results in faster data processing and effectively higher specificity and sensitivity because signals outside the window are blinded out.

RTs can be normalized using the iRT approach, if a spectral library is compiled from heterogeneous DDA runs or if the DDA runs used to generate the spectral library were generated with different gradient lengths as compared to the DIA runs 17, 20, 24. The iRT scale was initially developed for the simple scheduling of SRM assays. Later on the concept was adopted for the targeted analysis of DIA data 14, 17, 1. The iRT scale is defined on a set of 11 non‐naturally occurring peptides 24. Based on these 11 peptides the set of iRT anchor points can be extended to other peptides. This approach has been used to define iRT values for a set of peptides conserved in eukaryotic cells and was validated in several species 25. Targeted analysis of DIA data based on iRT prediction has been shown to improve sensitivity and speed of the analysis^1^. Furthermore, with iRT, spectral libraries can be generated from heterogeneous DDA runs, they can be transferred to other experiments and shared with other laboratories while retaining the advantages of RT prediction for the targeted analysis.

A number of software tools exist for the targeted analysis of DIA data among which are Spectronaut, OpenSWATH, Peak View and Skyline 26, 27. All of them make use of RT prediction.

The original iRT scale is based on a linear transformation over the whole gradient and does not consider local fluctuations or non‐linearities, which cannot be captured with a linear model. In this study, we made use of the high‐precision iRT which is generally applicable and was implemented in Spectronaut. Instead of using a linear regression, Spectronaut uses a segmented regression for the conversion of RT to iRT (spectral library generation) and iRT to RT (DIA data analysis) 17. The segmented regression approach makes use of many more than the 11 iRT anchor peptides (described in detail in Methods).

Here, we investigated how iRT precision influences the identification of peptides and precision as well as the reproducibility of quantitation. We, furthermore, systematically evaluate the influence of iRT precision on the transferability of spectral libraries. For this purpose, we simulated iRT inaccuracy and compared this to nine distinct experimental factors such as column length, temperature and mobile phase acid to measure their influence on iRT precision.

## 2 Materials and methods

### 2.1 Materials

Frozen HeLa cell pellets were purchased from Dundee cell products. Jurkat cell pellets were kindly provided by Dr. Thomas Uhlmann (Dualsystems AG, Schlieren). Iodoacetamide, tris(2‐carboxyethyl)phosphine, trifluoroacetic acid, formic acid, ammonium formate, ACN, HPLC water, ammonium bicarbonate and urea were purchased from SIGMA‐Aldrich. Trypsin sequencing grade was purchased from Promega. RapiGest was purchased from Waters.

### 2.2 Sample preparation

A 15‐cm dish of confluent cells was washed three times with PBS and then lysed by resuspension in 8 M urea and 0.1 M ammonium bicarbonate (to 1 μg/μL protein). For Jurkat cells, 10^6^ cells were collected by centrifugation and washed with PBS, then the pellets were resuspended in 8 M urea and 0.1 M ammonium bicarbonate (to 1 μg/μL protein). The lysates were reduced with 5 mM tris(2‐carboxyethyl)phosphine for 1 h at 37°C. Subsequently, the lysate was alkylated with 25 mM iodoacetamide for 20 min at 21°C. The lysate was diluted to 2 M urea and digested with trypsin at a ratio 1:100 (enzyme to protein) at 37°C for 15 h. The samples were spun at 20 000 × *g* at 4°C for 10 min. The peptides were desalted using C18 MacroSpin columns from The Nest Group according to manufacturer's instructions. After drying, the peptides were resuspended in 1% ACN and 0.1% formic acid. The Biognosys’ iRT kit, was added to all of the samples according to manufacturer's instructions (required for the DIA analysis using Biognosys’ Spectronaut).

### 2.3 High pH reversed phase fractionation

The HeLa digest was further fractionated using high pH reversed phase chromatography. 50 μg of the digest was adjusted to pH 10 using 0.2 M ammonium formate. Next, the sample was applied to a MicroSpin C18 column (The nest group). The peptides were eluted at 5, 10, 15, 20, 25 and 50% ACN in 0.05 M ammonium formate. Then the samples were dried and resuspended in 1% ACN in 0.1% formic acid. The peptide concentration was determined using a Spectrostar Nano (BGM Labware).

### 2.4 Mass spectrometric acquisition

As a reference setup, 2 μg of the samples was analyzed using a self‐made analytical column (75 μm x 40 cm length, fritted tip New Objective) packed with ReproSil‐Pur 120A C18‐AQ, 1.9 μm at 50°C on an Easy‐nLC 1000 connected to a Q Exactive mass spectrometer (Thermo Scientific) (if not specified else). The peptides were separated by a 2 h segmented gradient from 5 to 8% ACN in 2 min, next to 12% ACN in 9 min, next to 29% ACN in 94 min and to 34% ACN in 12 min with 0.1% formic acid at 300 nL/min, followed by a linear increase to 90% ACN in 2 min and 90% ACN for 8 min. For the 1, 2, 3, 4, 8 h runs a linear scaling of the segments was performed. For DDA MS runs, the “fast” method from Kelstrup was used with alterations as described in 28. The full scan was performed between 350 and 1650 *m/z*. Stepped collision energy was +/−10% at 25%. The DIA‐MS method consisted of a survey scan at 70 000 resolution from 350 to 1650 *m/z* (AGC target of 5×10^6^ or 120 ms injection time). Then, DIA windows were acquired (AGC target 3×10^6^ and auto for injection time, the DIA windows are described in Supporting Information Table‐1). Stepped collision energy was 10% at 25%. The spectra were recorded in profile mode. The default charge state for the MS2 was set to 4. The SWATH‐MS data was recorded on an Eksigent nanoLC connected to a Sciex Triple TOF 5600 mass spectrometer. 1 μg of the samples was analyzed on a self‐made analytical column (75 μm x 20 cm) packed with 3 μm Magic C18AQ medium (Bruker) at 50°C. The peptides were separated by a 2 h linear gradient from 5 to 35% ACN with 0.1% formic acid at 300 nL/min, followed by a linear increase to 98% ACN in 2 min and 98% for 8 min. The raw mass spectrometric data, the spectral library and the quantitative data tables have been deposited to the ProteomeXchange Consortium via the PRIDE partner repository with the dataset identifier PXD004188.

### 2.5 Mass spectrometric raw data analysis

DIA data was analyzed with Spectronaut 8, a mass spectrometer vendor independent software from Biognosys. The default settings were used for targeted analysis of DIA data in Spectronaut. In brief, RT prediction type was set to dynamic iRT and correction factor for window 1 (to linear with no extended calibration for the 11 iRT peptide‐based analyses). Mass calibration was set to local mass calibration (Supporting Information Fig. 1). Decoy generation was set to scrambled (no decoy limit)^2^. Interference correction on MS2 level was enabled. The FDR was estimated with the mProphet approach 28 and set to 1% at peptide precursor level. Protein inference, which gave rise to the protein groups, was performed on the principle of parsimony using the ID picker algorithm as implemented in Spectronaut 29. For the iRT noise experiments, the extraction for the extensive recalibration was set to 0.75 fraction of the gradient. The DDA spectra were analyzed with the MaxQuant Version 1.5.1.2 analysis software using default settings with the following alterations (minimal peptide length was 6). The identifications were filtered for 1% FDR on peptide and protein level. The DDA files were searched against the human UniProt fasta database (state 11.12.2014, 42 004 entries), and the Biognosys iRT peptides fasta database (uploaded to the public repository PRIDE). The peptide coefficients of variation (CVs) were calculated using the summed intensities of their respective fragment ions, the protein CVs were calculated based on the summed intensities of their respective peptides. When we use the word peptides in this study we refer to peptide precursors.

### 2.6 Extended iRT calibration set and spectral library generation

To generate an extended iRT calibration set, the following steps were performed. Highly accurate apex RTs for a large number of peptides derived from a HeLa digest were determined using DIA. DIA instead of DDA was used because apex RTs derived from DIA are more accurate (data not shown). First, a spectral library was generated from the DDA spectra using the classic 11 iRT peptides and linear regression in Spectronaut. Second, three linear DIA MS runs of HeLa lysate (linear from 0 to 40% B in 2 h) were analyzed using the above‐described spectral library and the classical 11 iRT peptide method. For this analysis in Spectronaut, a linear regression using the iRT peptides was chosen in the settings. Then, a report was generated on peptide precursor level containing the modified peptide sequences and the apex RTs. The mean and SD of the RTs for peptide precursors were calculated for the triplicate (only precursors identified in all three acquisitions were included). The peptide precursor identifications were binned into 20 equally sized RT bins from the first to the last eluting peptide precursor. Per bin, the 80% of the peptide precursors with the lowest SD were selected (to minimize outliers from, e.g., in source fragmentation) to result in the final extended iRT recalibration set (the 11 iRT peptides were all kept). Finally, a linear regression iRT ∼ RT_mean_ based on iRT‐pep b (iRT=0) and iRT‐pep l (iRT=100) was performed as described in 24 and iRT values were assigned to all peptides using this regression. The two peptides iRT‐pep b and iRT‐pep l were originally used to define the iRT scale. The resulting HeLa iRT calibration set was loaded into Spectronaut (21 155 anchor peptides (including the original iRT peptides), Supporting Information Table 2, public repository PRIDE).

To generate the final spectral library, DDA data was acquired, searched with MaxQuant and a spectral library was generated using the spectral library generation functionality of Spectronaut which made use of the previously generated HeLa iRT calibration set. The default settings for spectral library generation were used. In brief, segmented regression to determine iRT in each run was used as described below. iRTs were calculated as median iRTs across all DDA runs. Fragment ions <300 *m/z* and >1800 *m/z* as well as fragment ions with less than three amino acid residues were not considered. Fragment ions with neutral losses were included. A peptide precursor was added to the spectral library if minimally three fragment ions could be detected in the MS2 spectrum. Maximally six, most intensive, fragment ions were kept.

### 2.7 iRT ∼ RT and RT ∼ iRT segmented regression and extraction window width estimation as implemented in Spectronaut

Both, during spectral library generation and for targeted analysis of DIA data, Spectronaut performs a segmented regression if enough data points are available (min. 200 for spectral library generation, min. 50 for targeted analysis of DIA). For spectral library generation, the set of anchor points correspond to the overlap of the DDA results with internal data base of iRT calibration anchor points in Spectronaut. For this publication, the internal database consisted of the 21 155 anchor points derived from the HeLa cell line sample (described above). For DIA analysis, the anchor points correspond to an automatic first pass analysis always performed by Spectronaut with a linear regression and very wide extraction windows.

For the segmented regression, the data points are split up into bins along the RT (spectral library generation) or iRT (DIA analysis) dimension. Each bin contains max(*n*/40, 20) data points, where n is the total number of anchor points. The total number of bins is max(*n*/40, 20)×2 – 1 because the bins are overlapping by half. For each bin, a robust Theil‐Sen regression is performed. The median *x*‐value of this bin is used as reference for this bin. The reference *y‐*value is calculated using the Theil–Sen regression. After calculating the reference *x*‐*y* points for all bins, the points are connected using lines between them. At the edges the values are linearly extrapolated.

For the estimation of the window width, the same set of iRT/RT anchor points is split into bins along the iRT dimension (min. 30 anchor points otherwise a fix window is applied). This time the bin size of the bins is chosen *n*/log2(*n*), where n is the total number of anchor points. The bin overlap is *n*/log2(*n*)‐1, this means the bin is “sliding” by one index over the anchor points. The reference *x*‐value for a bin is calculated as the average iRT. The reference *y*‐value is calculated as (*w* + *q*)*0.5, where *w* is the median chromatographic width of all detected peaks in this bin and *q* is the 75th percentile of the difference between measured RT and predicted RT (estimated as described above). These reference *x*‐*y* points are fed into the same segmented regression as described above with bin size m/10, where m is the number of reference *x*‐*y* points. The window width is chosen two times the predicted value of this regression.

## 3 Results

### 3.1 High‐precision iRT in DIA

**Figure 1:**
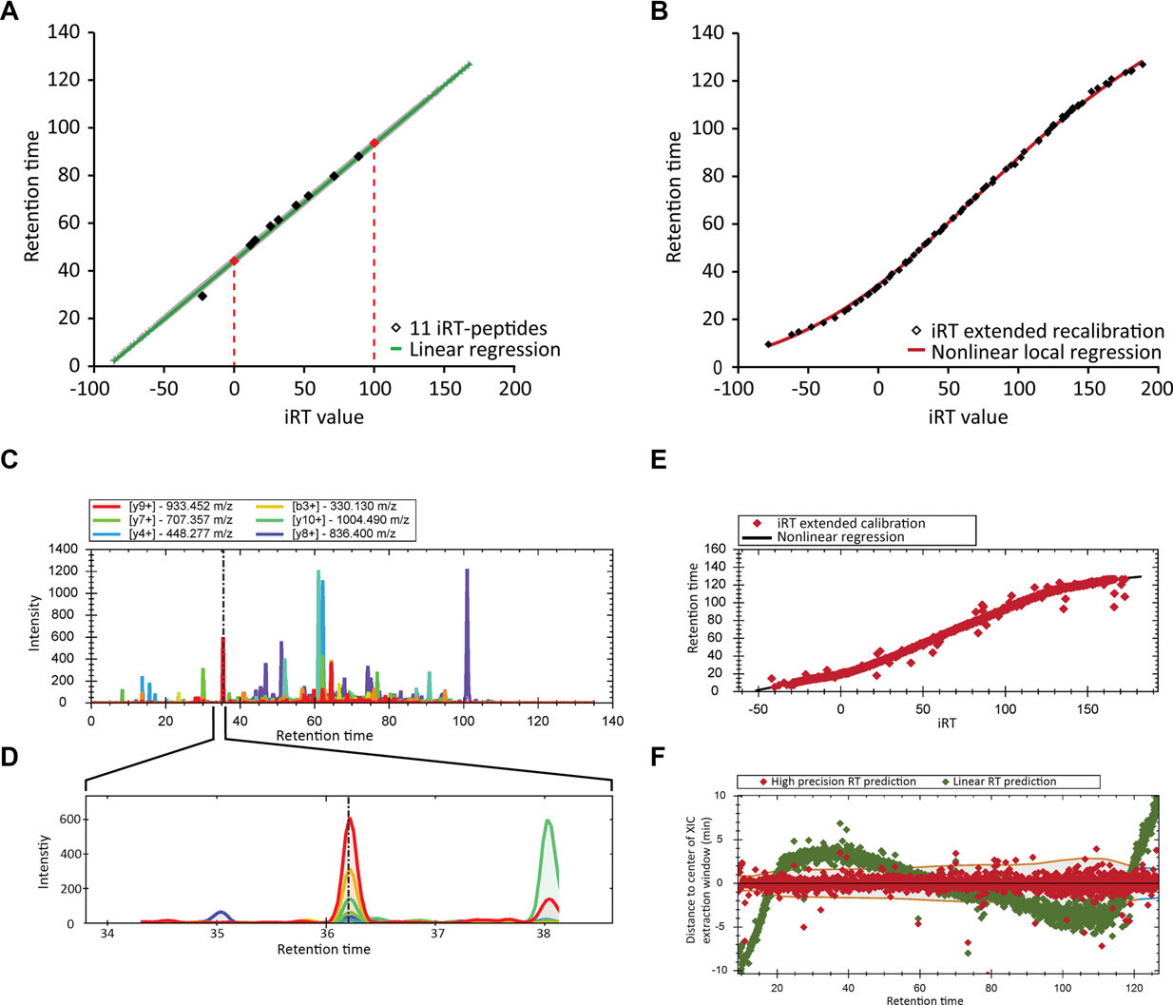
Introduction of high‐precision iRT determination. (A) The 11 originally published iRT peptides were used to extend the iRT set of anchor points with three DIA MS runs (iRT‐pep b = 0, iRT‐pep l = 100, red‐dotted lines). (B) A spectral library with high‐precision iRT values were generated by segmented regression of DDA data based on an extended iRT recalibration set of 21 155 peptides. (C) In the whole XIC of a DIA run, multiple peak groups are observed. (D) The usage of high‐precision iRT prediction enables the focus of the targeted analysis on a small fraction of the total XIC leading to improved sensitivity and data quality. (E) In DIA, the iRT values of a set of high‐quality peptide identifications can be used for a segmented regression of RT as a function of iRT (visualization as in the Spectronaut software). (F) The distance to the middle of the estimated RT extraction window for a DIA‐MS measurement when using a segmented regression (red) and as a comparison when using a linear regression (green) is shown (visualization as in the Spectronaut software).

The iRT system was initially developed for SRM. With 11 iRT peptides it was mostly used with linear gradients and has a limited RT precision. In order to augment iRT to very high precision, we had to extend the set of iRT anchor points. For this purpose, we measured our sample with a linear gradient in DIA triplicates. We performed a linear regression using the original 11 iRT peptides (24 and methods) to obtain an iRT calibration set with 21 155 peptides (including the original iRT peptides) and assigned iRT (Fig. 1A, Supporting Information Table 2). These anchor points were imported in Spectronaut. As of release 8, Spectronaut holds a large database of iRT anchor points generated in a similar fashion, extending the presented human iRT set. The presented set can serve as a basis for segmented, local regression also in other software packages. This large set of anchor points can be used during the spectral library generation process to deliver very high‐precision iRTs (Fig. 1B and methods).

For DIA‐MS, the iRTs of the spectral library are used to predict the RT and perform a targeted analysis of the peptide signals. The more precise the predicted RT values are the smaller the extraction window and the higher the quality of the analysis (Fig. 1C and D). It minimizes potential ambiguity of similar peptides species (for example of similar precursor mass and similar fragmentation patterns), by zooming for the analysis only into an XIC of a usually 0.5–2% of the gradient (for high‐precision iRT). The usage of thousands of high quality iRT values is well suited for the recalibration of the segmented, non‐linear acquisitions (Fig. 1E and methods). Further, the window size dynamically changes and adapts to the less precise parts of the gradient (Fig. 1F and methods). Outlier points in terms of iRT residuals (Fig. 1E and F) are likely to be false discoveries estimated at 1% (*q*‐value ≤ 0.01).

### 3.2 Effect of iRT precision

The above‐described approach results in high‐precision iRT. When the experimental conditions change as in the reuse of a spectral library in another laboratory with a different setup, the precision would likely decrease. Therefore, we investigated the effect of the iRT precision on the performance of DIA acquisitions. Also, we wanted to compare high‐precision iRT to not using any RT prediction at all (full ion trace extraction) as well as the original iRT based on only 11 anchor points and linear regression. For this study, a HeLa spectral library was generated on the reference setup based on DDA acquisitions of whole sample (14 MS runs) and high pH reversed phase fractionation (six MS runs). The HeLa spectral library contained 77 296 peptide precursors, 58 601 peptide sequences of 5412 protein group identifications. (Supporting Information Table 3, public repository PRIDE.)

**Figure 2:**
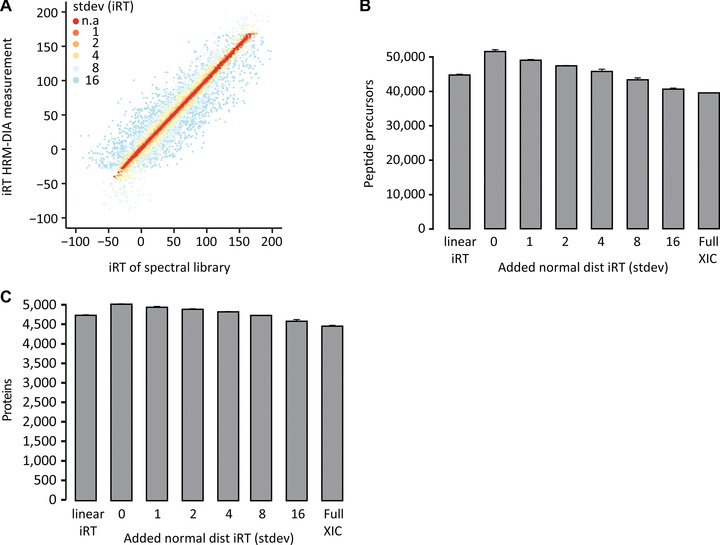
Isolated effect of iRT precision on identification. (A) Imprecise iRT values were simulated by adding normally distributed iRT noise to the reference spectral library (0, 1, 2, 4, 8 and 16 iRT SD). The modified spectral libraries were used for the targeted analysis of a HeLa in a technical triplicate DIA. Additionally, the DIA runs were analyzed using no RT prediction (Full XIC) and linear regression based on the 11 original iRT peptides. The peptide (B) and protein (C) identifications were calculated for the analyses with the modified spectral libraries. The mean identifications with SD were plotted.

To simulate a decrease in iRT precision, we used the spectral library with high‐precision iRT and added normally distributed iRT noise to it (iRT SD). These variants were applied to a HeLa technical triplicate DIA on the reference setup (Fig. 2A and Supporting Information Table 4 public repository PRIDE). Except for the full ion trace extraction and the linear 11 iRT peptide analysis, default Spectronaut settings were used, which means that the extraction window width is automatically chosen (see methods).

The analysis using the unmodified high‐precision iRT spectral library revealed an absolute median delta iRT of 0.53 (0.27% of the gradient). The median extraction window width was 4.1 min (3.4% of the gradient). A maximal identification of 53 659 peptide identifications corresponding to almost 5000 protein groups (4871 exactly) was achieved in a single 2 h Q Exactive DIA measurement.

We could monitor a significant drop in peptide identifications upon iRT inaccuracy introduction up to 23% (high‐precision iRT to iRT SD 16). Full XIC performed similar to iRT SD 16 (Fig. 2B). The protein identifications dropped by roughly 500 (Fig. 2C). The classic workflow with the 11 iRT peptide anchor points performed in the range of iRT SD 6. The automatically, dynamically selected extraction width in Spectronaut increased in a linear fashion with iRT noise (Supporting Information Fig. 2 and Table 5).

Additionally, we wanted to assess the benefit of high‐precision iRTs in linear DIA runs. Therefore, we performed linear DIA runs with HeLa samples and analyzed the data either with the classical 11 iRT peptides linear or the segmented regression. The segmented regression resulted in 18% more identified precursors in the linear DIA runs, this difference was significant (*t*‐test, two sample, two tails, *p*‐value = 7.6 × 10–5) (Supporting Information Fig. 3).

### 3.3 Quantification analysis of iRT precision

**Figure 3:**
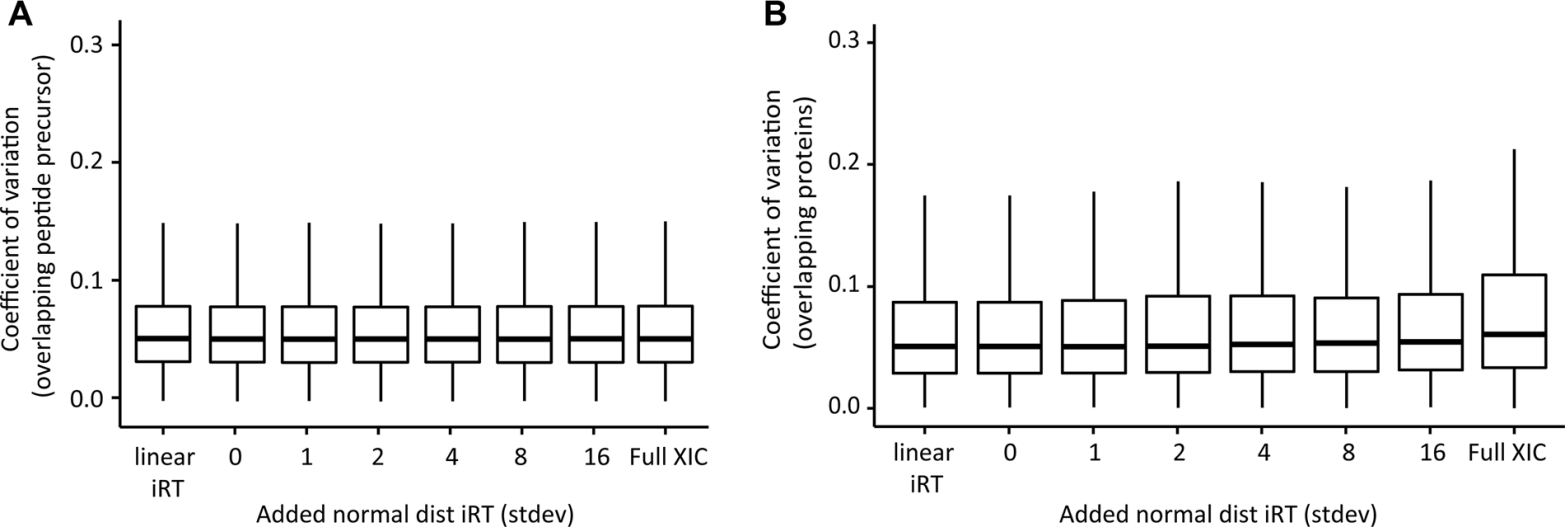
Effect of iRT precision on quantification. CVs for the analyses of HeLa in technical triplicate DIA when using spectral libraries with decreasing iRT precision, when using no RT prediction at all (full XIC) and when using only the original 11 iRT peptides and linear regression. The mean identifications with SD were plotted. (A) CVs of the overlapping peptides were calculated based on normalized intensities. (B) CVs for the overlapping protein identifications were calculated based on summed peptide intensity per MS run.

Next, we wanted to see the effects of increasing iRT inaccuracy on the precision of quantitation. We calculated the CVs of only the overlapping peptides for all the analyses (28 908 overlapping peptides) (Fig. 3A). The CVs of the identified peptides remained stable (median around 5%).

For proteome profiling studies comparing different states, the differential abundance of proteins is of foremost interest. We calculated the CVs for 3640 overlapping proteins from all analysis (Fig. 3B). Only a slight increase (relatively 7% increase for CVs of full XIC compared to the unmodified high‐precision iRT spectral library) in CVs was observed likely due to decreasing coverage of peptides per protein.

### 3.4 Effect on reproducibility

A major advantage of DIA over DDA mass spectrometry was shown to be the high reproducibility of identification and quantification 17, 25, 30, 31. We analyzed the impact on targeted DIA search using the iRT noise modified spectral libraries for the profiling of peptides.

**Figure 4:**
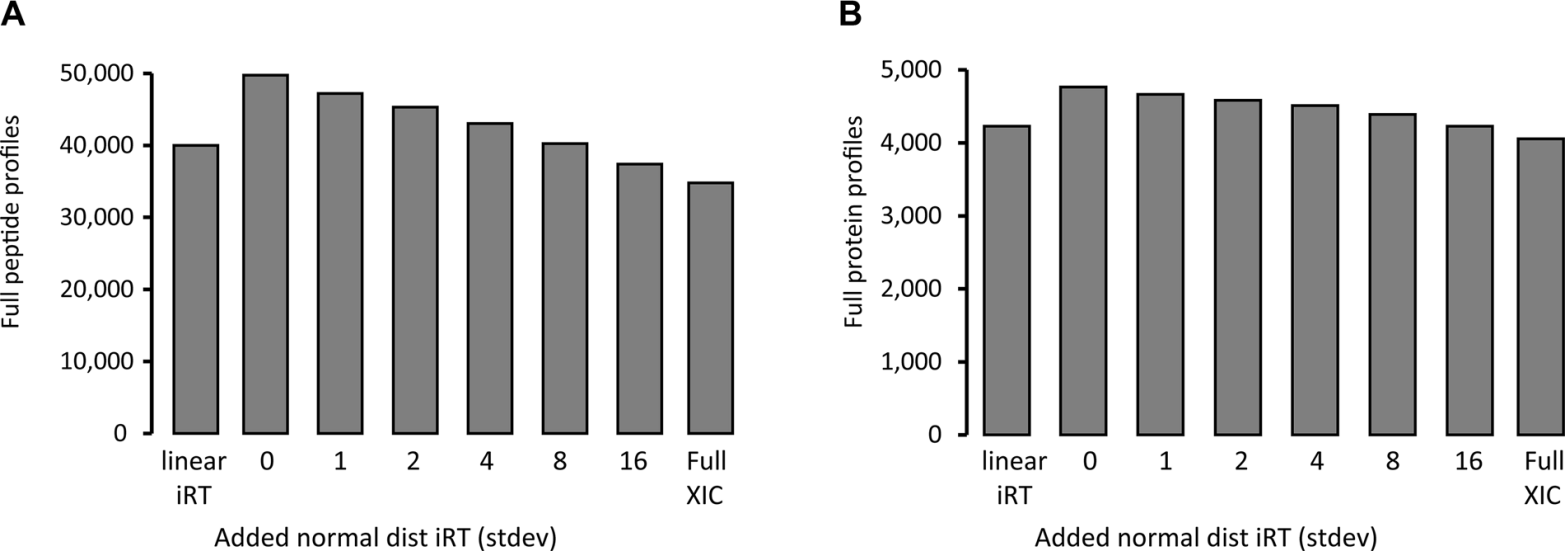
Effect of iRT precision on reproducibility of identification. Reproducibility for the identifications for a HeLa in technical triplicate DIA was analyzed when using spectral libraries with decreasing iRT precision, when using no RT prediction at all (full XIC) and when using only the original 11 iRT peptides and linear regression. (A) The set of peptides that were identified without missing values were counted. (B) The set of protein identifications that were identified without missing values were counted.

For this purpose, we counted the number of consistent identifications of peptides for DIA triplicates. With increasing iRT noise in the spectral libraries, the set of completely identified peptides (identified in all three replicates) dropped by 23% (Fig. 4A). The set of completely profiled proteins dropped by 16% (Fig. 4B). To verify whether our findings are relevant for other DIA setups, we performed the same analysis on a classical SWATH‐MS acquisition with a triple TOF 5600 instrument using a linear gradient 14. The spectral library was performed on this instrument using 24 DDA MS runs of a HEK‐293 sample. We observed a similar dependence on identification and a retaining of high quality of quantification for CVs of 16 335 overlapping peptides and improvement to the linear iRT approach (Supporting Information Fig. 4).

### 3.5 Experimental factors influencing iRT precision

iRT precision was very high, if the spectral library was generated with the identical experimental setup as the DIA runs were. We were interested how the variation in various experimental factors affects iRT. This is especially relevant for the transferability of spectral libraries to other laboratories.

**Figure 5:**
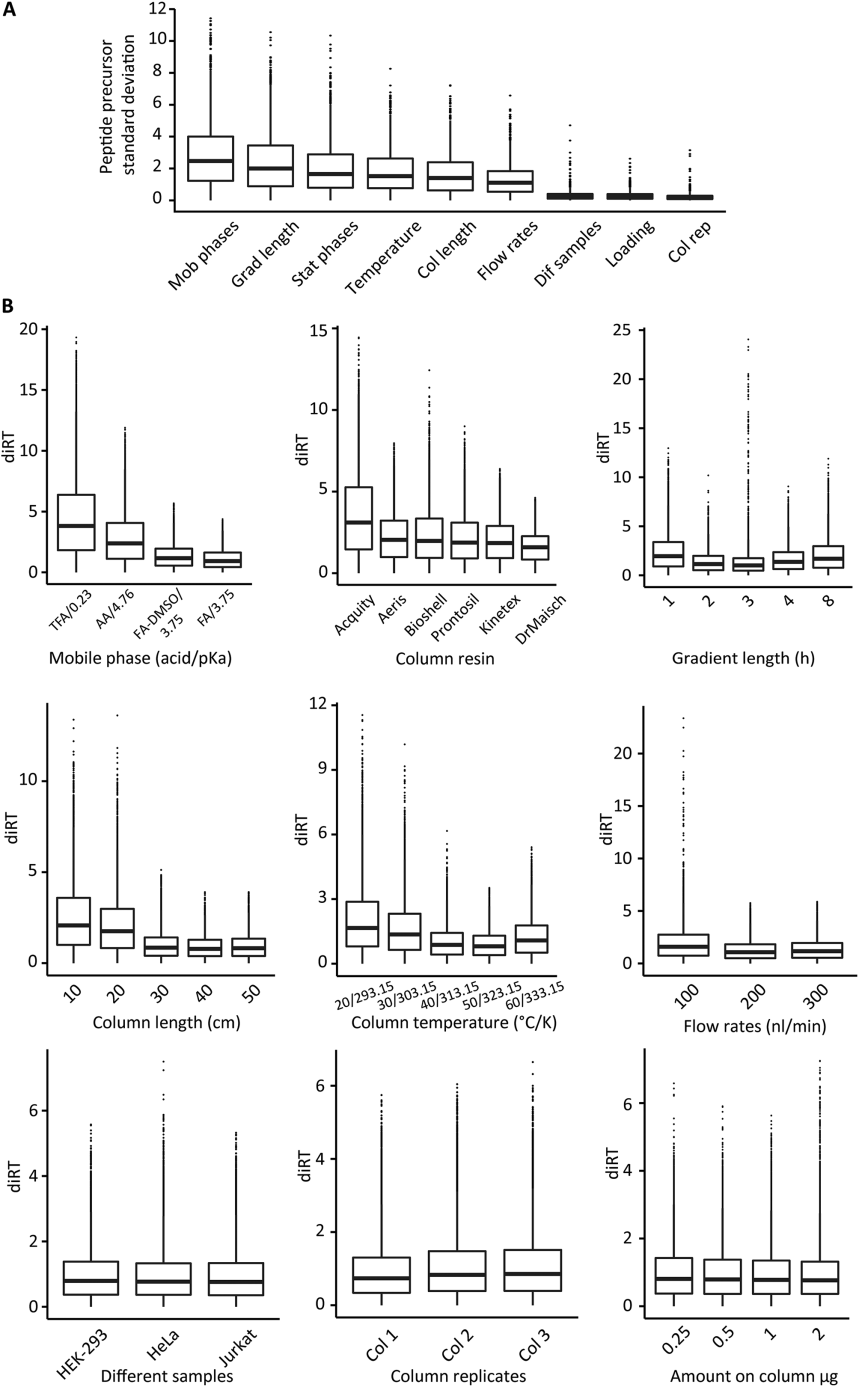
Experimental factors influencing iRT precision. (A) Experimental iRT values were determined for nine practically relevant experimental factors: stationary and mobile phases, column and gradient length, column temperature, flow rates, column replicates, sample types and sample on column amount. The SD of the iRT values for overlapping peptides of the most diverging conditions per factor were calculated. (B) The deviation in iRT to the reference spectral library was determined for all conditions within the experimental factors.

We varied nine important experimental factors resulting in 30 different DIA measurements in total. The DIA data were analyzed using the high‐precision iRT approach. First, influence of the varying experimental factors was investigated by comparing the iRT SDs of the two most extreme conditions per factor (Fig. 5A). These SDs can be related to the analysis of Fig. 2 to estimate its effect. The mobile phase acid had the largest influence (TFA to formic acid, Fig. 5B). Surprising to us was the relatively large influence of the gradient length (1 to 8 h, Fig. 5B). Not surprisingly, the stationary phase had a relatively large influence, i.e., the third largest. A small influence was observed for different human cell lines, column replicates and sample loading. A change of the mobile phase acid resulted in an iRT SD of less than 3. When comparing this to Fig. 2B this would roughly correspond to adding iRT noise with SD of 3 and would result in more than 45 000 peptides identified (about 90% of the maximum) which is still higher than typically achieved with a single DDA run.

### 3.6 Influence on iRT precision within the specific factors

Within one varying factor, we were also interested in the specific conditions that show the largest iRT deviations.

The iRT values of the peptides were compared to the respective mean iRT of all 30 conditions and a delta iRT was calculated (Fig. 5B). The TFA condition showed the strongest iRT deviation of all investigated factors. For the stationary phases the Acquity beads showed the largest deviations. We could also see a larger deviation than expected when going from a 20 cm to a 30 cm column. All other influences were either minor or behaved linearly.

### 3.7 Influence of changing the experimental condition on identification of peptides

We also looked at the cumulative effect of changed condition and increased iRT accuracy on identification of peptides when compared to the reference condition (see methods) (Supporting Information Fig. 5). The non‐linear regression was used for the data analysis. The usage of a 10 cm column resulted in the strongest reduction of identifications. As expected, changing the gradient length had a strong influence in identifications. The 8h acquisitions resulted in over 86% coverage of the spectral library used. For the different column and gradient length, the different stationary phases, the DIA methods were adapted to keep data points per peak constant, which also contributed to the differences in identifications in these acquisitions.

## 4 Discussion

Targeted analysis of DIA is a powerful method for the comprehensive, reproducible and precise quantitation of dozens to hundreds of samples 17, 18, 30. A spectral library is a crucial part of such an analysis 17, 18, 20, 22. Three major factors influence the performance of a spectral library: the selection of contained peptides, the selection of fragment ions and relative intensities thereof and the peptide RT information. In this study, we were interested in the added value of the RT information contained in a spectral library. We were mainly interested in two aspects: The influence of iRT precision on the analysis and the influence of experimental factors on the precision of iRT and hence on the transferability of spectral libraries.

By extending the set of iRT anchor points to thousands a robust segmented regression can be used to convert RT to iRT (spectral library generation) or iRT to RT (targeted DIA data analysis). This has the advantage that heterogeneous shotgun runs can be combined to generate spectral libraries and for DIA data analysis smaller XIC extraction windows can be used which positively influences the sensitivity and speed of analysis. This holds true also for linear gradients, as demonstrated in Supporting Information Figs. 2 and 4.

We found a significant correlation between iRT precision and the number of identifications. When going from high‐precision iRT to no RT prediction at all the number of peptide precursor identification dropped up to 30% (11% on protein level). Also when comparing to the classical iRT using linear regression and only the original 11 iRT peptides the identifications dropped by 13% (6% on protein level) for non‐linear DIA runs. Notably, even 40 000 peptide precursors (4500 protein groups) identified without RT prediction are a high number for a 2 h measurement on a Q Exactive mass spectrometer 32.

When looking at quantitation the influence of iRT was minor. For the proteins the slight increase in CVs can be explained with decreasing peptide coverage of the proteins. We concluded that high coverage improves quantitation on protein level, even if mainly low abundant signals are added. Generally, quantitation was of high quality with median CVs below 10%.

Given the importance of the spectral libraries, it is natural to think about sharing these spectral libraries to avoid generating them over and over again [20, 22]. The transferability of spectral libraries is a pivotal aspect, especially when changing an experimental condition between spectral library generation and DIA measurement. A possible reason for unequal experimental factors could be distinct instrumentation in two different laboratories. When looking into the influence of experimental factors, we found that the most influential factor on iRT precision out of nine factors tested is the mobile phase acid (i.e., most likely the pH resulting from the acid). This was not unexpected, however, the scale of influence was found to be relatively small: the median iRT SD was only ∼2.5 which corresponds to roughly 1.3% of the total gradient length. The second most influencing factor was the gradient length and the third most influencing factor was the stationary phase. This suggests that it is better to keep the gradient length constant and use other means to increase the coverage of the spectral library such as sample pre fractionation or measuring the samples most distantly related in the experiment. Three factors had almost no influence on iRT precision: varying the sample (three different human cell lines), exchanging the column or varying the sample load.

High‐precision iRT, as supported in the software Spectronaut, increases the sensitivity and speed of targeted DIA analysis. Generally, the quality of identification (45 000 peptide precursors) and quantitation (CVs of 40 000 peptide precursors at median of about 6%) is very high when comparing to state of the art literature, even when experimental conditions change between the spectral library generation and DIA acquisition 33, 34, 35. Nevertheless, for the transferability of spectral libraries it is advisable to keep the experimental conditions as similar as possible to the setup used to generate the spectral library. Especially, a change of the mobile phase acid should be avoided.

A possible solution to improve RT prediction for spectral libraries from a clearly distinct setup might be to use an in‐run trained RT prediction algorithm and predict the delta rather than the absolute RT. The data points needed for the training could be easily generated from a first pass analysis.

In general, our findings are valuable for RT alignment algorithms and RT prediction. This data set can be used as a resource to develop in silico RT prediction algorithms for different experimental conditions.

*L.R. and R.B. designed the project. R.B. carried out the measurements. R.B. did the data analysis. L.R. and R.B. designed the acquisition method. T.G. and O.M.B wrote the software. R.B. and L.R. wrote the paper. L.R. supervised the project*.

*The authors R.B., T.G., O.M.B. and L.R. are employees of Biognosys AG (Switzerland). Spectronaut is a trademark of Biognosys AG*.

## Supporting information

As a service to our authors and readers, this journal provides supporting information supplied by the authors. Such materials are peer reviewed and may be re‐organized for online delivery, but are not copy‐edited or typeset. Technical support issues arising from supporting information (other than missing files) should be addressed to the authors.

Supporting Information

Supporting Information

Supporting Information

Supporting Information

Supporting Information

Supporting Information

## Footnotes

1 Bernhardt, O. M., Selevsek, N., Gillet, L. C., Rinner, O., Picotti, P., Aebersold, R., and Reiter, L. (2012) Spectronaut A fast and efficient algorithm for MRM‐like processing of data independent acquisition (SWATH‐MS) data. Proceedings of the 60th ASMS Conference on Mass Spectrometry and Allied Topics, 2012, Vancouver, BC, Canada.

2 Bernhardt, O. M., Bruderer, Gandhi, T., Miladinović, S. M., Bober, M., Ehrenberger, T., Rinner, O., and Reiter, L. (2014) General guidelines for validation of decoy models for HRM/DIA/SWATH as exemplified using Spectronaut. Proceedings of the 62nd ASMS Conference on Mass Spectrometry and Allied Topics, 2014, Baltimore, MD, USA.
