# Supplementary figures and images for: High‐precision iRT prediction in the targeted analysis of data‐independent acquisition and its impact on identification and quantitation
Source: Proteomics. 2016 Jun 28;16(15-16):2246–56. doi: 10.1002/pmic.201500488 (PMC5094550; doi:10.1002/pmic.201500488)

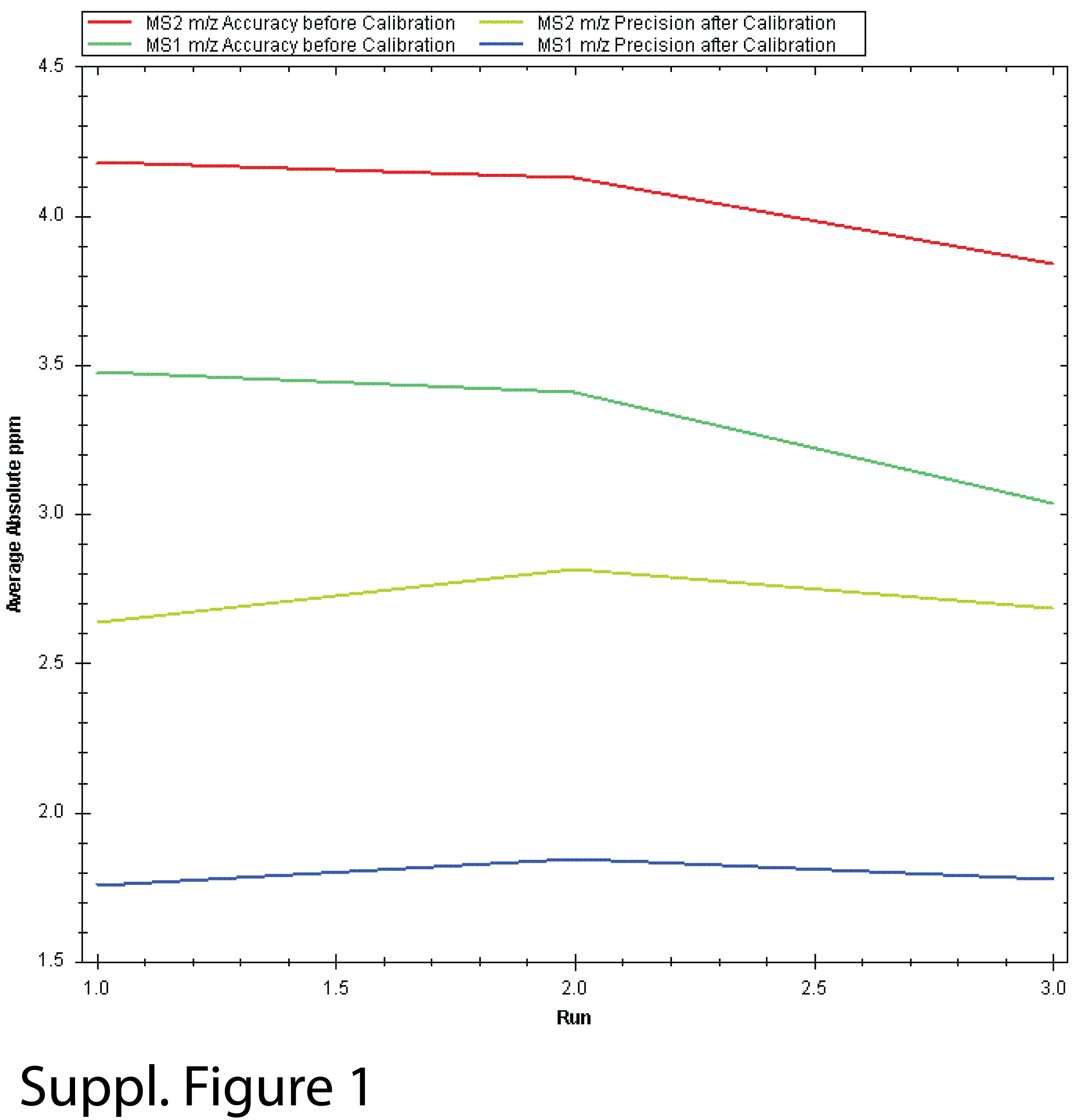

Supplement: Supplementary file 2 — Supporting Information [file PMIC-16-2246-s002.tif]

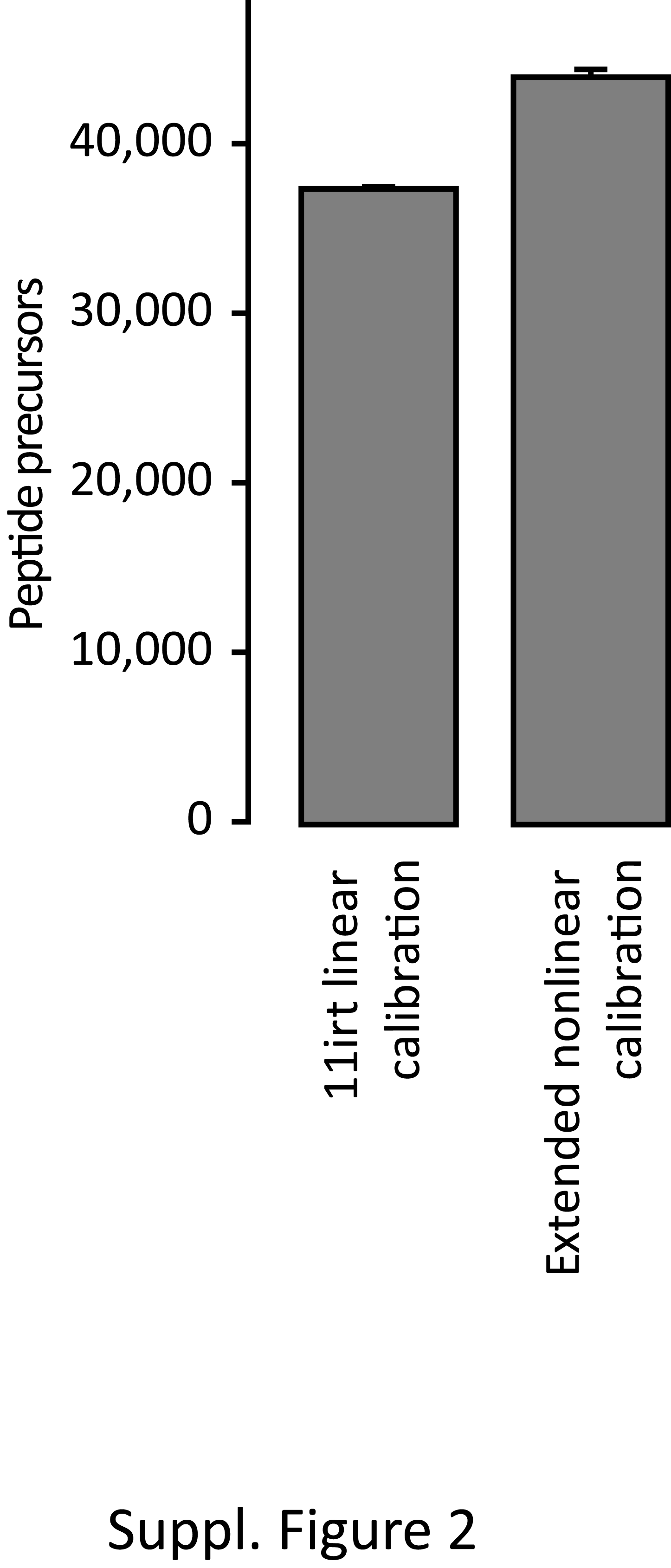

Supplement: Supplementary file 3 — Supporting Information [file PMIC-16-2246-s003.tif]

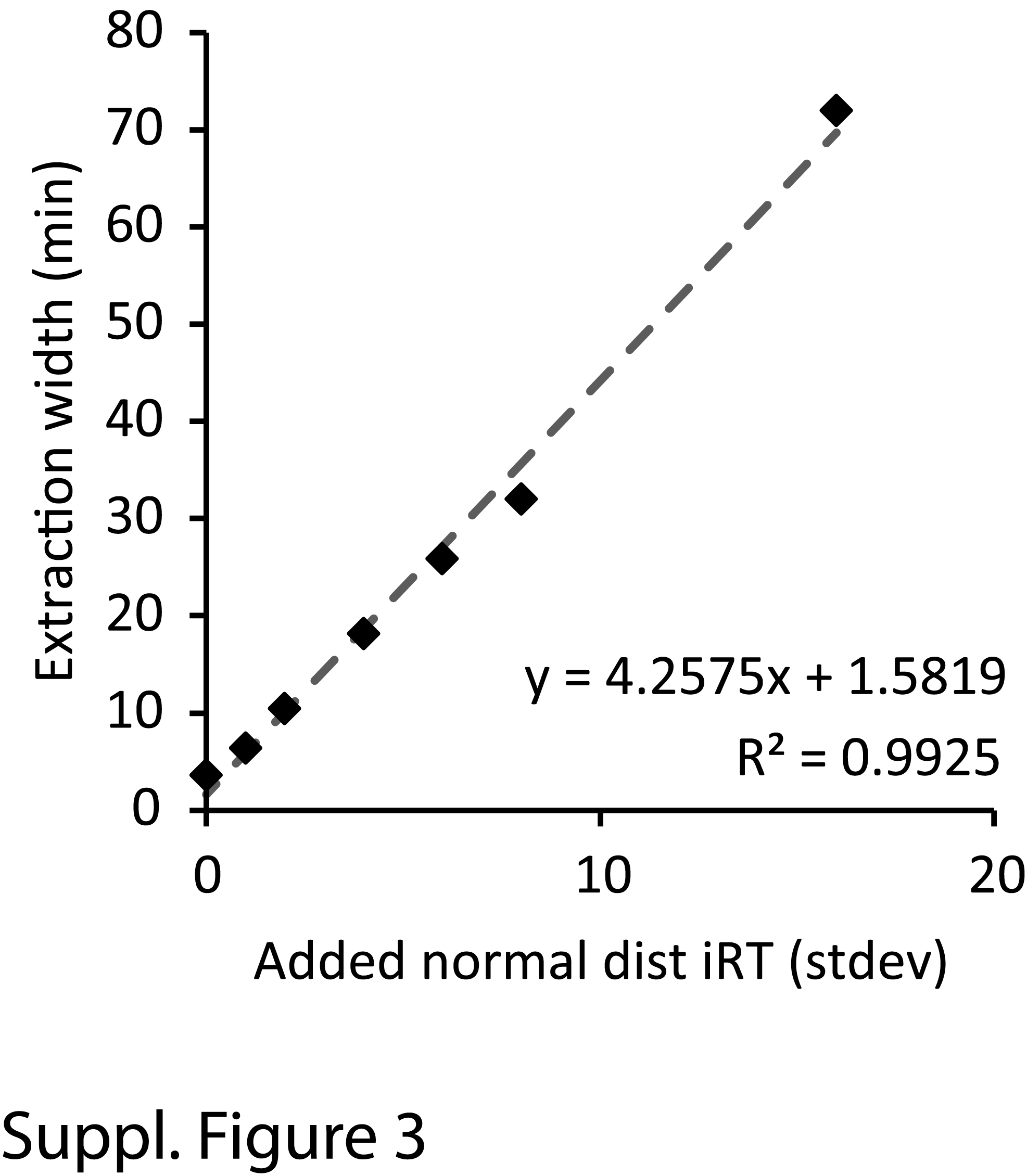

Supplement: Supplementary file 4 — Supporting Information [file PMIC-16-2246-s004.tif]

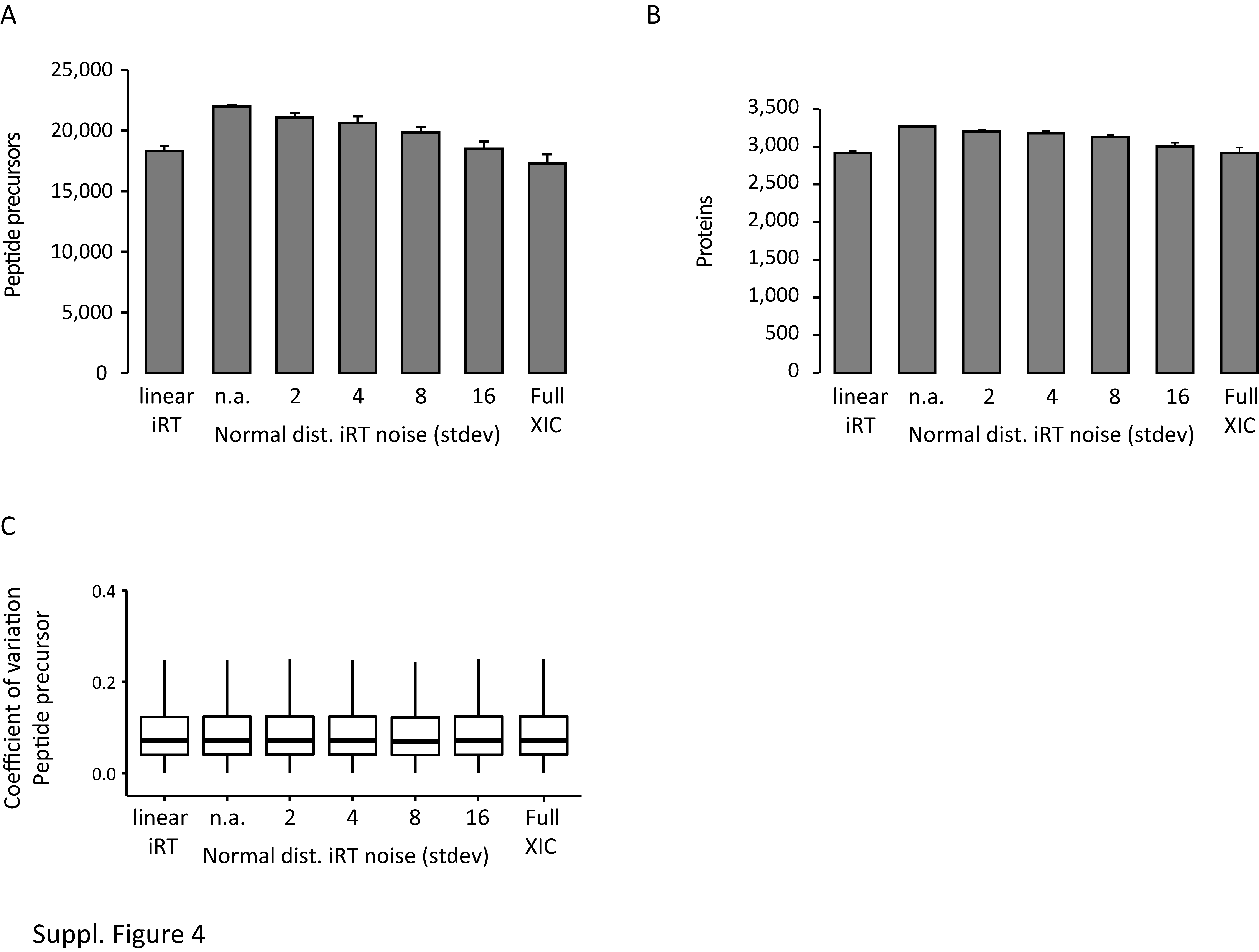

Supplement: Supplementary file 5 — Supporting Information [file PMIC-16-2246-s005.tif]

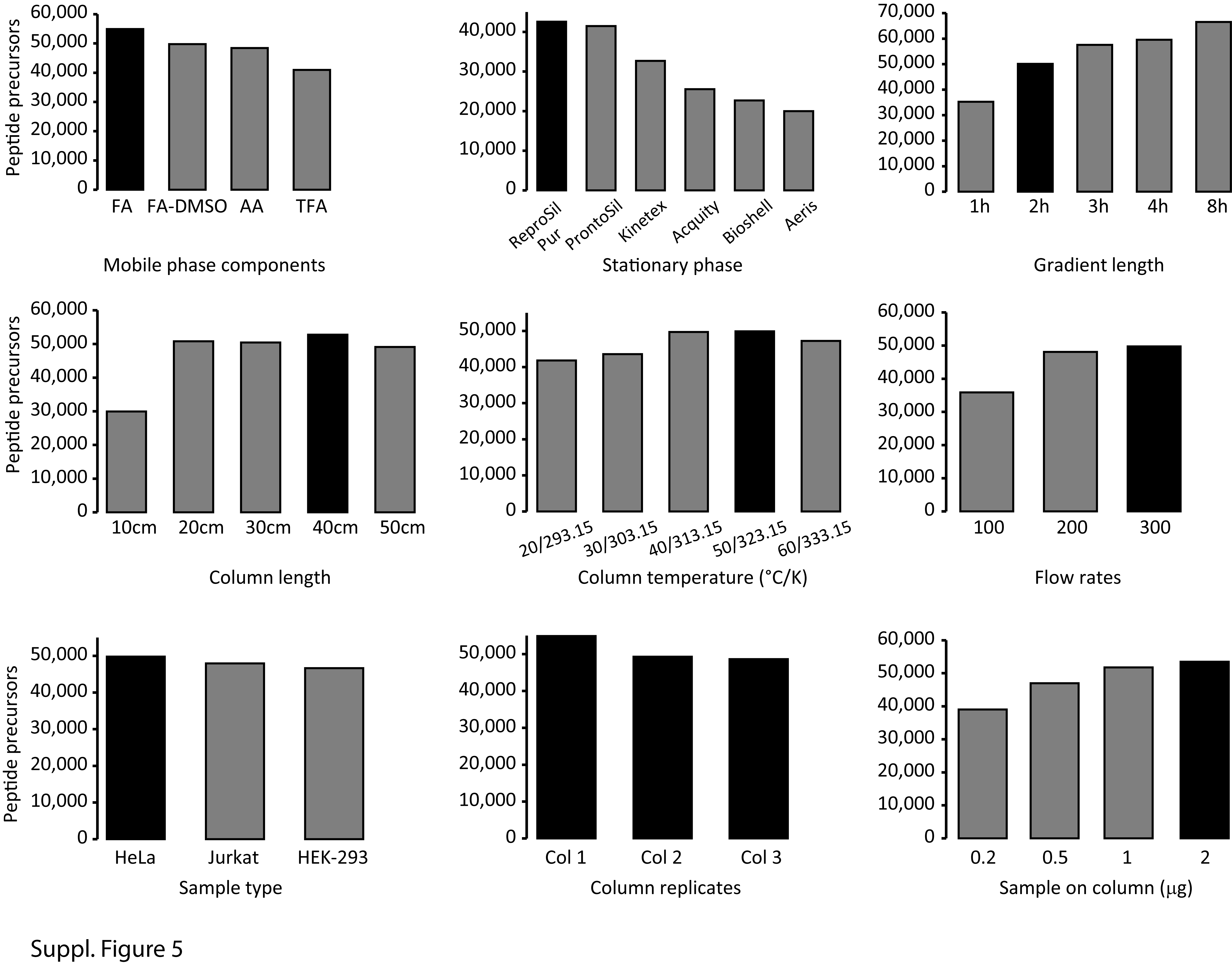

Supplement: Supplementary file 6 — Supporting Information [file PMIC-16-2246-s006.tif]
